# Clean Joints, Clean Planet: A Review of Literature on Sustainable Practices in Orthopaedic Joint Replacements

**DOI:** 10.7759/cureus.112414

**Published:** 2026-07-10

**Authors:** Amey M Borse, Janam Merchant, Vaishnavi S Bhave, Nahush M Borse, Vijay Patil

**Affiliations:** 1 Department of Trauma and Orthopaedics, Northern Care Alliance NHS Foundation Trust, Oldham, GBR; 2 Department of Trauma and Orthopaedics, Wrightington, Wigan and Leigh NHS Foundation Trust, Wigan, GBR; 3 Medicine, Independent Researcher, Oldham, GBR; 4 Department of Orthopaedics and Traumatology, Maharashtra University of Health Sciences, Islampur, IND

**Keywords:** arthroplasty, carbon footprint, green orthopaedics, implant recycling, joint replacement, net zero healthcare, surgical waste management, sustainability

## Abstract

The healthcare sector generates significant global greenhouse gas emissions, and the orthopaedic operating theatre is among the most resource-intensive environments within any hospital. Hip and knee arthroplasty produce considerable waste and carbon emissions with each procedure. This review evaluates global sustainable practices adopted for hip and knee joint replacement surgeries and identifies practical opportunities to reduce environmental impact while preserving patient safety and quality of care. A narrative review rather than a systematic review of literature of major databases was performed, and evidence concerning energy efficiency, reusable instruments, waste management and implant production and recycling was analysed. Operating theatres consume significantly more energy than other hospital areas, but occupancy-based ventilation can reduce heating, ventilation and air conditioning (HVAC) energy consumption, as well as the use of light-emitting diode (LED) lighting and shutdown protocols. Sterilisation accounts for the majority of emissions attributed to reusable instruments, while the rationalisation of surgical trays and reusable surgical gowns and drapes can also reduce CO₂ emissions. Each arthroplasty generates a significant amount of waste. The implant manufacturer heavily contributes to procedural emissions, and the global recycling of explanted implants could yield substantial environmental and financial savings. Sustainable arthroplasty is both achievable and cost-saving. Energy conservation, reusable instruments, better waste segregation, implant recycling, and evidence-based patient selection can help reduce the environmental and economic burden of joint replacement surgery. Furthermore, collaboration between surgeons, healthcare institutions and the healthcare industry is essential to balance high-quality patient care with sustainability.

## Introduction and background

The environmental impact of healthcare delivery has become a subject of increasing scrutiny as health systems worldwide confront the dual imperatives of providing high-quality clinical care and reducing their contribution to the climate crisis. The healthcare sector is estimated to be responsible for approximately 4-5% of global greenhouse gas emissions, a footprint that, if it were a single nation, would rank it among the largest emitters in the world [1]. Surgical services occupy a disproportionate share of this burden, as the operating theatre is one of the most resource- and energy-intensive environments within any hospital, owing to its demanding requirements for ventilation, lighting, single-use consumables and instrument reprocessing [1,2]. Addressing this issue requires understanding key sustainability concepts, such as life-cycle assessment (LCA), which evaluates the environmental footprint of a product from raw material extraction to disposal, and carbon dioxide equivalent (CO₂e), a standard unit for measuring carbon footprints.

Orthopaedic surgeries and joint replacements in particular lie at the heart of this challenge. Hip and knee arthroplasties rank among the most frequently performed elective procedures globally, and the demand continues to rise in step with population ageing and growing prevalence of osteoarthritis. More than 3.6 million knee replacements were undertaken worldwide in 2023, and approximately one million hip replacement procedures were performed each year [3]. Each individual procedure is estimated to generate in and around 190 kg of CO₂e, and this is the quantity which is compatible with the emissions produced by a single domestic flight [3,4]. When this figure is multiplied across the millions of arthroplasties which are performed annually, the aggregate environmental cost is quite considerable. The waste generated is similarly substantial; a single total knee arthroplasty produces on average 14 to 17 kg of waste material, the overwhelming majority of which is currently consigned to incineration or landfill rather than being recovered or recycled [5].

These pressures have prompted various health institutions to adopt formal decarbonisation commitments. In the United Kingdom, the National Health Service (NHS) has pledged to reach net zero carbon emissions across the wider scope of its activities and supply chains by the year 2045. This is a target which places direct obligations upon the surgical specialities to examine and reform their practices [6]. Orthopaedics, as a high-volume speciality with a well-characterised and substantial carbon footprint, is uniquely positioned both to contribute to and to benefit from this transition. Crucially, many of the interventions which are proposed to reduce environmental harm, such as rationalising instrument trays, adopting reusable gowns and drapes and improving waste segregation, also offer meaningful financial savings which align the goals of environmental sustainability and financial responsibility [7].

This review examines the published evidence on sustainable practices in hip and knee arthroplasty by synthesising current knowledge along the four principal domains, such as maintaining energy efficiency in the operating theatre, the use of reusable surgical instruments, proper waste management and adequate implant production and recycling. By implementing such a strategy, it seeks to identify practical, evidence-based opportunities to reduce the environmental impact of joint replacement surgery without compromising patient safety or quality of care.

## Review

Materials and methods

A narrative review of the published literature was undertaken to identify and synthesise evidence concerning sustainable practices in joint replacement surgery. Three electronic bibliographic databases, namely PubMed, Embase and Google Scholar, were searched for relevant articles published between 1st January 2000 and 1st June 2025. The search strategy employed combined keywords and terms, including sustainability, green orthopaedics, carbon footprint, arthroplasty, life cycle assessment and waste management; these were used individually and in combination.

The review concentrated on literature addressing one or more of the four principal thematic domains: energy efficiency within the operating theatre, the environmental impact of reusable surgical instruments and gowns, waste segregation and management associated with arthroplasty, and the production, longevity and recycling of orthopaedic implants.

Studies were eligible for inclusion if they were peer-reviewed primary research or review articles, published in English between 1st January 2000 and 1st June 2025, that addressed the environmental impact, resource use, waste, cost or sustainability of hip or knee arthroplasty within one or more of these domains. Studies were excluded if they did not address environmental or sustainability outcomes relevant to arthroplasty, were not available in full text, or were limited to conference abstracts, editorials or opinion pieces without supporting data. Within these boundaries, both primary research studies and relevant review articles were considered, and sources were selected purposively for their relevance to the four domains rather than through an exhaustive, protocol-driven search

Data concerning carbon emissions, energy and resource consumption, waste output, recycling potential and associated economic outcomes were extracted and synthesised thematically. This was a selective, narrative synthesis rather than a systematic review: no formal screening protocol, risk of bias appraisal or quantitative pooling of data was undertaken. The databases searched, date range, search terms and thematic scope are summarised in Table 1.

**Table 1:** Literature search approach

Parameter	Detail
Databases searched	PubMed, Embase, Google Scholar
Date range	2000-2025
Search terms	sustainability; green orthopaedics; carbon footprint; arthroplasty; life cycle assessment; waste management (used individually and in combination)
Thematic domains	Operating-theatre energy efficiency; reusable instruments and textiles; waste management; implant production and recycling
Study types	Primary research studies and review articles
Evidence synthesis	Narrative (qualitative)

Sustainable strategies in joint replacement surgery

The evidence on sustainable practice is considered below across four principal domains: energy use, reusable instruments, waste management and implant production and recycling, followed by the contribution of patient selection and education. Table 2 provides an overview of these strategies and their reported environmental and economic impact, each of which is examined in turn below

**Table 2:** Summary of sustainable strategies in hip and knee arthroplasty and their reported environmental and economic impact HVAC, heating, ventilation, and air conditioning; LED, light-emitting diode; THA, total hip arthroplasty; TKA, total knee arthroplasty; GIRFT, Getting It Right First Time.

Domain	Key strategies	Reported environmental and economic impact	References
Energy use in the operating theatre	Demand-controlled (occupancy-based) ventilation; LED lighting; end-of-list shutdown checklists; alcohol-based hand rubs	Theatres use up to 4× more energy than other hospital areas; ventilation control reduces HVAC energy by 20-40%; LED and shutdown protocols reduce energy by a further ~25%	[1,2,4]
Reusable instruments and textiles	Rationalisation of surgical trays; reusable cotton gowns and drapes	Sterilisation accounts for ~90% of reusable-instrument emissions; tray rationalisation saves up to 524 kg CO₂ per theatre per year; reusable textiles cut emissions by up to 65%	[2,3]
Waste management	Segregation at the point of generation; recycling of recovered metals	THA generates ~14.46 kg and TKA ~17.16 kg of waste per procedure, with <5% recycled; Sweden recovers ~60 tonnes of metal annually (~US$15 million) following cremation	[5,8]
Implant production and recycling	Recycling of explanted implants; selection of durable, long-lived implants	Implant manufacture contributes ~28% of procedural emissions; global recycling of explanted implants could save ~£184 million annually	[3,8]
Patient selection and education	Evidence-based non-operative management; adherence to GIRFT	Avoids low-value procedures, reducing emissions and cost at source	[9,10]

Energy Use in the Operating Theatre

The operating theatre is the single most energy-intensive area within the hospital, consuming as much as four times more energy per unit floor area than other clinical areas [1,2]. The principal driver of this consumption is the heating, ventilation and air conditioning (HVAC) system, which sustains most high air exchange rates and, in many authentic theatres, the laminar flow conditions traditionally employed to minimise the risk of periprosthetic joint infection. Because these systems frequently operate continuously, including outside of operating hours, they represent a substantial and often unnecessary source of energy expenditure.

A consistent finding across the literature is that occupancy-based or demand-control ventilation offers considerable scope for energy reduction. By automatically lowering air handling rates when a theatre is unoccupied and restoring full ventilation. When clinical activity resumes, such systems have been shown to reduce HVAC energy consumption by between 20 and 40% without compromising the sterility of the operative field [4]. Further savings are achievable through relatively straightforward measures: the replacement of conventional theatre lighting with light-emitting diode (LED) alternatives, together with the introduction of a structured end of list shutdown checklist to power down non-essential equipment. Such practices have been reported to reduce energy by a further 25% or thereabouts [4]. The adoption of alcohol based hand rubs in place of traditional surgical hand scrubbing additionally reduces water consumption, contributing to the broader sustainability of theatre practices while maintaining standards of surgical asepsis. 

Reusable Surgical Instruments

An LCA quantifies a product's environmental impact across its whole life span, from raw-material extraction through manufacture and use to disposal. For surgical instruments, such analyses consistently demonstrate that the dominant contribution to their environmental footprint arises not from manufacture but from reprocessing. Sterilisation encompassing cleaning, decontamination and autoclaving accounts for approximately 90% of the total emissions attributable to reusable instruments [2]. This finding has important implications, as it indicates that the environmental performance of reusable instruments is closely tied to both the efficiency of the sterilisation process and the volume of instruments processed.

This means that rationalising surgical trays is a particularly effective intervention. Orthopaedic procedures are frequently supported by large instrument sets, many components of which are seldom or never used. By auditing tray contents and reducing them to the instruments genuinely required for a given procedure, the mass of metal requiring sterilisation, along with the associated energy and emissions, can be reduced substantially. Such tray optimisation has been reported to reduce carbon dioxide emissions by up to 524 kg per operating theatre per year [2,3]. Compatible benefits or rights from the choice of surgical textiles: the transition of single-use disposable gowns and drapes to reusable cotton equivalents has been shown to reduce associated emissions by as much as 65% while also reducing the volume of clinical waste generated [2].

Waste Management

In orthopaedics, joint replacement surgery is a significant generator of clinical waste. Total hip arthroplasty produces approximately 14.46 kg of waste per procedure and total knee arthroplasty approximately 17.16 kg, of which less than 5% is currently recycled; this low recycling rate reflects the handling of surgical and wider clinical waste streams, of which arthroplasty waste forms a part [5]. The remainder is predominantly directed towards high-temperature incinerators or landfill, both of which carry substantial carbon and financial costs. A considerable proportion of this waste comprises materials including packaging, single-use drapes and metal that are in principle amenable to recovery.

The evidence indicates that improved segregation of waste at the point of generation, combined with dedicated recycling of implant grade and other metals, can markedly reduce both the environmental burden and the cost of waste disposal [5]. Effective segregation ensures that materials suitable for recycling or lower-impact treatment are not inadvertently consigned to the most carbon-intensive and expensive disposal streams. The scale and economic value of metal recovery are well illustrated by Swedish experience: since the 2016 regulation made the recycling of all metal collected after cremation mandatory, Sweden has recovered approximately 60 tonnes of metal annually with a reported net value of around $15 million, equivalent to roughly £11 million [8].

Implant Production and Recycling

The manufacturer of orthopaedic implants constitutes a major component of the overall carbon footprint of arthroplasty, contributing approximately 28% of the total emissions associated with a joint replacement procedure [3]. This suggests that the energy-intensive processes which are involved in extracting, refining and machining the high-grade metal alloys from which modern metal implants are constructed. The end-of-life management of these implants represents a very substantial and largely untapped opportunity for environmental and economic gain.

The metal implants which are explanted during revision surgery, or which are recovered following cremation, contain significant quantities of recyclable material. The consensus is that the global recovery and recycling of explanted orthopaedic implants could yield savings in the region of around £184 million annually alone in the USA and Europe [8]. Realising this potential requires the good establishment of robust collection, processing and recycling pathways which are still yet to be developed completely. Parallelly, the selection of implants designed for durability and longevity also reduces the likelihood of revision surgery, thereby avoiding the considerable environmental and economic cost associated with repeat procedures [3,8].

Patient Selection and Education

A complementary and frequently overlooked dimension of sustainable arthroplasty concerns the appropriateness of surgery itself. The most environmentally and economically efficient operation is one that is avoided where it would confer limited clinical benefit. The promotion of evidence-based, non-operative management strategies, including physiotherapy, weight optimisation, and analgesia for patients who are unlikely to benefit substantially from surgical intervention, can reduce the number of procedures performed and, consequently, their associated environmental impact [9]. Adherence to established quality improvement frameworks such as the Getting It Right First Time (GIRFT) programme supports this aim by enhancing the consistency, efficiency and appropriateness of surgical care, thereby reducing unwarranted variation and avoidable procedures [10].

Economic and environmental impact

Sustainable surgical practice is quite necessary from an economic perspective. The healthcare institutions, depending on the scale and the combination of measures adopted, may realise annual savings which may range from approximately £11,000 to £116,000 through the implementation of green operating theatre practices [7,8]. The substitution of reusable linens for disposable equivalents alone can reduce associated operating theatre expenditure by up to 65% [2]. Also, a reduction in the volume of waste consigned to incineration translates directly into lower disposable cost while energy efficiency measures reduce utility expenditure over time [7]. More importantly, these interventions confer benefits beyond the balance sheet by reducing global pollution and the emissions associated with healthcare delivery; they also reduce public health harms which could arise from environmental degradation, mandating sustainable surgery within a broader framework of population health [1].

Policy context: the NHS clinical waste strategy

The policy context in the United Kingdom shows the scale of challenge as well as the structure of coordinated response. NHS trusts in England produce around 156,000 tonnes of clinical waste each year, which is projected to rise with an upcoming increase in arthroplasty procedures, simultaneously increasing carbon emissions and cost. So in response, a 10-year national strategy has been adopted with the aim of transforming NHS waste management into a system that is safer, more efficient and environmentally sustainable, in support of the NHS commitment to net zero carbon by 2045 [6]. This strategy is organised around six interrelated priorities. The first, data, seeks to improve the accuracy of this data collection and reporting to at least 95%, enabling evidence-based decision-making. The second, workforce, requires every NHS trust to employ a dedicated and appropriately trained waste manager with clearly defined responsibilities and opportunities for professional development. The third, compliance, targets the achievement of proper waste segregation broadly apportioned between high temperature incineration, alternative treatment and offensive waste streams through staff training and monitoring. The fourth, commercial, aims to standardise contracts and improve procurement in order to reduce waste management costs per tonne. The fifth, infrastructure, calls for investment in resilient onsite and regional waste treatment facilities. The sixth, sustainability, sets ambitious targets for the reduction of carbon emissions arising from waste through optimised resource use and adoption of innovative low-impact practices. Together these priorities provide a robust, structured framework within which the speciality-level interventions described in this review can be embedded and scaled [11].

Recommendations for sustainable practice

Drawing on the evidence reviewed, several practical recommendations emerged for orthopaedic units seeking to reduce their environmental impact. Operating theatres should adopt occupancy-based ventilation systems to minimise energy consumption during periods of inactivity. Surgical instrumentation should be standardised on the surgical trays, kept to the minimum necessary, which will reduce the burden of sterilisation. Waste segregation and recycling within the operating theatre should be improved with particular attention to the recovery of metals and other recyclable materials. Orthopaedic implants should be selected with regard to longevity, and, where possible, compatibility with recycling programmes should be kept in mind in order to reduce both revision rates and end-of-life waste. Finally, sustainability should be incorporated into medical education, training curriculum and institutional leadership and policy programmes, ensuring that environmental considerations become an integral component of surgical decision-making rather than an optional addition [6,9]. Crucially, these measures should be implemented collaboratively involving infection prevention and control teams, theatre and nursing staff, sterilisation and decontamination services, and procurement teams so that any change is safe, feasible and clinically acceptable. Throughout, sustainability interventions must be adopted without compromising infection control, implant longevity and outcomes or patient safety, which remain the absolute priorities.

Barriers and challenges to implementation

Notwithstanding the strength of the evidence, the translation of sustainable practise into routine clinical care faces significant obstacles. A significant obstacle among these is the cultural resistance which is frequently underpinned by concerns regarding infection control. The historical association between laminar airflow ventilation, single-use products and the prevention of periprosthetic joint infection has fostered a degree of caution that can impede the adoption of reusable instruments and modified ventilation strategies even where the evidence indicates that patient safety is not compromised [6,9]. A second barrier is a general lack of awareness of the environmental impact of surgical practice, which is compounded by the upfront financial and logistical demands associated with transitioning to more sustainable systems. The initial investment required for reusable processing infrastructure or theatre enhancements, for example, may deter institutions despite the prospect of long-term savings [6]. Third, the complexity of medical supply chains and the regulatory requirements governing medical devices and infection prevention render systemic change difficult to implement and slow to take effect [9]. Overcoming these barriers will require not only robust evidence but also sustained leadership, financial incentives and cultural change across the speciality.

Limitations

This review has several limitations that readers should consider when interpreting its findings. First, it was conducted as a narrative rather than a systematic review; no formal screening protocol, risk-of-bias appraisal or quantitative pooling of data was undertaken, and sources were selected purposively, which introduces a risk of selection bias. Second, the search was limited to three databases and to English language literature only, so relevant evidence indexed elsewhere or reported in other languages may have been missed. Third, much of the available evidence and many of the quantitative estimates cited here, including carbon emissions, waste and cost figures, derive from a small number of studies conducted in specific health systems (predominantly the United Kingdom, with additional data from Sweden and elsewhere) and rely on life-cycle analyses. These figures should therefore be regarded as indicative rather than precise. Moreover, healthcare systems differ substantially in their waste and recycling policies, energy sources, procurement practices and regulatory environments, so estimates of environmental and economic impact may not generalise directly between countries or currencies. Finally, sustainable surgical practice is a rapidly evolving field, and as new data emerge, they are likely to supersede current data estimates. Further to these findings, a more comprehensive review is needed to check global sustainability practices for hip and knee replacement surgeries.

## Conclusions

Sustainable practice in hip and knee arthroplasty is both achievable and financially advantageous. Energy-conserving measures within the operating theatre and the use of reusable instruments and surgical drapes and gowns offer a substantial reduction in carbon emissions at cost while preserving the quality and safety of patient care. Appropriate segregation of waste and the recycling of explanted implants further reduce the environmental and economic burden of joint replacement surgery, addressing the impact of surgery at multiple points along the care pathway. Beyond the operating theatre, patient education and adherence to established quality improvement frameworks such as the GIRFT programme may help to avoid procedures that are unlikely to give substantial benefit. Realising the full potential of these strategies will require sustained commitment and close collaboration between orthopaedic surgeons, healthcare institutions and industry partners working together to reconcile the delivery of excellent patient care with the imperative of environmental sustainability.
